# Commentary: Bioceramics and Scaffolds: A Winning Combination for Tissue Engineering

**DOI:** 10.3389/fbioe.2017.00015

**Published:** 2017-03-08

**Authors:** Eric Denes, Guislaine Barrière, Evelyne Poli, Guillaume Lévêque

**Affiliations:** ^1^I.ceram, Limoges, France

**Keywords:** alumina ceramic, porous ceramics, biomaterials, sternum, biocompatible materials, osteointegration

We read with interest the review published by Baino et al. (2015) about bioceramics and scaffolds. We would like to add some information as we produce a porous alumina ceramic (CERAMIL^®^) which present almost all the criteria that characterize an ideal scaffold as presented in table 1 of the article. Converse to the assertion that porous alumina is only used in the fabrication of orbital implants, our ceramic is widely implanted as vertebra cages and gap filling during opening wedge high tibial osteotomy. It has been recently implanted for the replacement of a tumor sternum.

The ceramic used is an alumina porous one processed thanks to a patented process [Patent FR2823674 (2006)].

Our technic seems to be in accordance to every point listed by the authors:

*Geometry*: several shapes are designed such as cubes, roof tiles, cylinders, trapezoidal parallelepiped, spheres, and complex one such as the sternum with holes for stitching. 3D complex shapes can be designed and small bones can easily be done (Figure 1).

**Figure 1:**
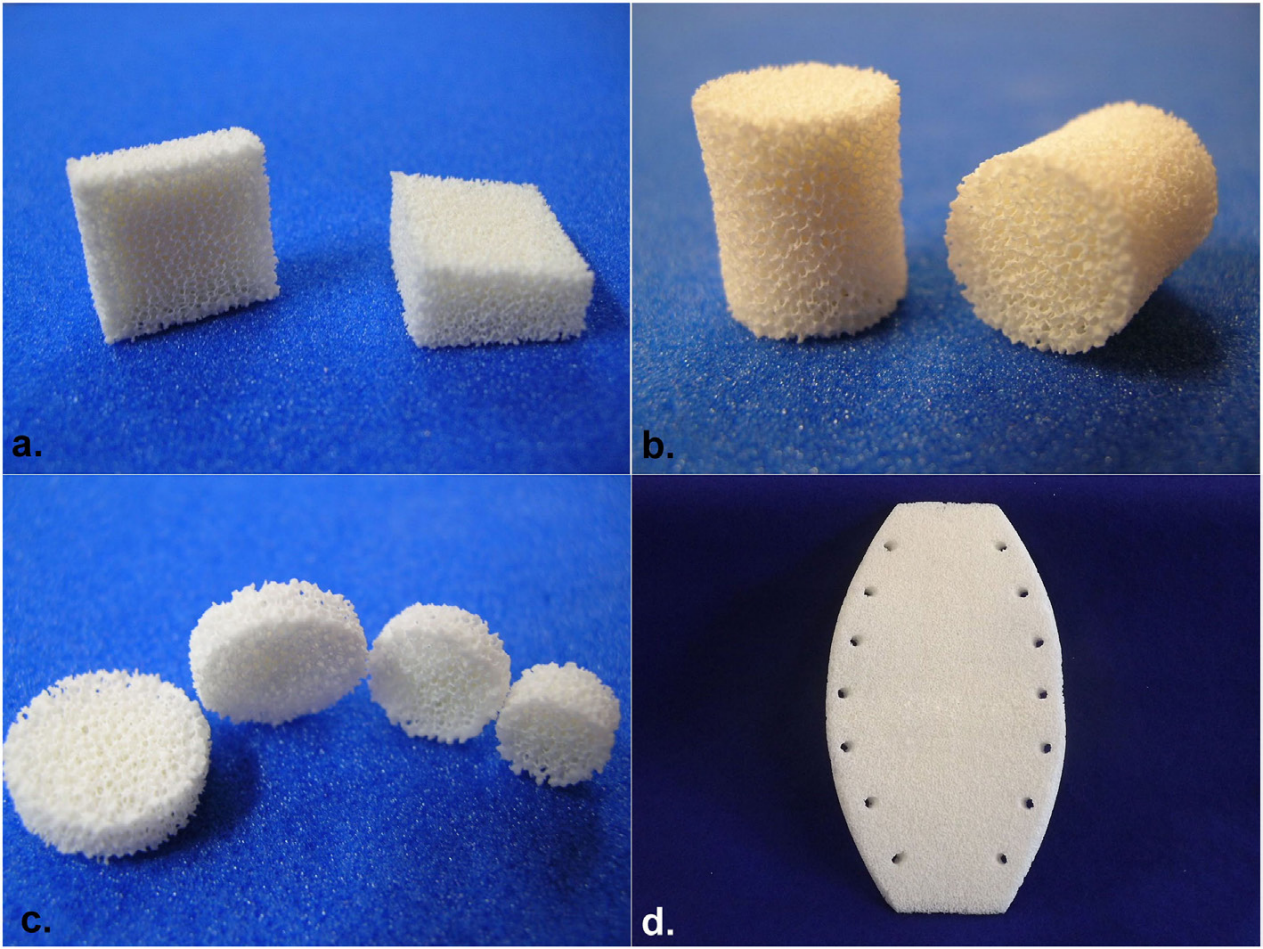
**(A)** Corporectomy cervical bloc, **(B)** intersomatic cylinder, **(C)** trepan hole filling pellets, and **(D)** sternal implant.

*Bioactivity*: the porous characteristic (see below) allows a rapid attachment of osteoblasts to the ceramic, and its integration has been studied by histopathology showing in-growth bone cell in pores. This characteristic leads to long-term bonding between ceramic and surrounding bone.

*Biocompatibility*: more than 5,000 implantations have been performed with vertebra cages, and the long-term follow-up does not show any case of local or systemic toxic effect. Studies are ongoing to analyze activity of bone cell in contact with the ceramic. Furthermore, alumina ceramic are classified as inert with no interaction with the surrounding tissues (Patel and Gohil, 2012; Baino et al., 2015).

*Chemical and biological stability*: as said by the authors, alumina has a very good bioinertness and good long-term mechanical properties without degradation (Baino et al., 2015).

*Porous structure*: with the technic used to fabricate our ceramic, the size of the interconnected pores is ranging from 100 to 900 μm with a vast majority of pore of about 600 to 900 μm. Moreover, all the pores are interconnected, without dead-end. This structure allows colonization with bone cells and thus the stability of the ceramic in the bone (Bignon et al., 2003; Hing, 2005; Lew et al., 2012). Moreover, pore’s size ranging from 600 to 1,250 μm seems to be the ones that allow the best colonization by bone cells (Bignon et al., 2003).

*Mechanical competence*: the ceramic possess a mechanical compressive resistance superior to 20 MPa. This resistance is superior to the bone one (1–7 MPa as defined by the French Society for Research in Orthopaedic Surgery & Traumatology). The resistance is proofed *in vivo* as it is used without any problem as a gap filling during opening wedge high tibial osteotomy. Moreover, clinical trials conducted for over 15 years, using implants of the range CERAMIL^®^, confirmed the consistency of the selection made. For example, Finiels (2004) observed a good mechanical stability and a bone fusion using porous alumina ceramic.

*Biological properties*: as previously said, alumina ceramic is inert, so there is no release of ions or other substance.

*Fabrication*: CERAMIL^®^ process allows us to tailor pieces that can fit bone defect such as those seen in tumor or complex infection bone surgery. All implants are proposed in many sizes in order to allow the surgeon to choose the right one during surgery, depending on the amount of tissues removed or based on the modification needed.

*Commercialization*: the ceramic we proposed is already commercialized in several countries (France, Italy, and Czech Republic) and is approved by ANSM (French National agency of medicine products safety). CERAMIL products possess the CE marking.

We would like to add another characteristic that is particularly of interest for an implant which is its resistance to infection. We showed that the amount of adherent *Staphylococcus aureus* strains is lower on alumina structure than on other classical material used for joint prosthesis and that the biofilm formation was lower on this material in comparison to polyethylene, titanium, or stainless steel (unpublished data). It seems to be confirmed *in vivo* as only 1 tibial osteotomy wedge has been infected among more than 5,000 implanted devices.

In conclusion, our porous alumina ceramic seems to possess all the characteristics listed by Baino et al. required to be a good scaffold. We wanted to let the readers know that such a ceramic is not used only in the fabrication of orbital implants but that it is already implanted for other clinical indication. Researches are still ongoing to improve the performances of this ceramic and to produce new shapes in order to help surgeons to restore bone structures in patients suffering of bone infection or bone tumors.

## Author Contributions

ED, GB, EP, and GL wrote, edited, and approved the manuscript. GL manufactured ceramic parts.

## Conflict of Interest Statement

ED, GB, EP, and GL are employed by I.ceram, which produces the porous alumina ceramic CERAMIL^®^.
